# Short-Term Effect of Cigarette Smoke on Exhaled Volatile Organic Compounds Profile Analyzed by an Electronic Nose

**DOI:** 10.3390/bios12070520

**Published:** 2022-07-13

**Authors:** Silvano Dragonieri, Vitaliano Nicola Quaranta, Enrico Buonamico, Claudia Battisti, Teresa Ranieri, Pierluigi Carratu, Giovanna Elisiana Carpagnano

**Affiliations:** 1 Respiratory Diseases Unit, Department SMBNOS, University of Bari, 70121 Bari, Italy; vitalianonicola.quaranta@asl.bari.it (V.N.Q.); enrico.buonamico@policlinico.ba.it (E.B.); c.battisti1@studenti.uniba.it (C.B.); teresa.ranieri@uniba.it (T.R.); elisiana.carpagnano@uniba.it (G.E.C.); 2 Internal Medicine “A.Murri”, University of Bari, 70121 Bari, Italy; pierluigi.carratu@uniba.it

**Keywords:** electronic nose, volatile organic compounds, breath analysis, smoking

## Abstract

Breath analysis using an electronic nose (e-nose) is an innovative tool for exhaled volatile organic compound (VOC) analysis, which has shown potential in several respiratory and systemic diseases. It is still unclear whether cigarette smoking can be considered a confounder when analyzing the VOC-profile. We aimed to assess whether an e-nose can discriminate exhaled breath before and after smoking at different time periods. We enrolled 24 healthy smokers and collected their exhaled breath as follows: (a) before smoking, (b) within 5 min after smoking, (c) within 30 min after smoking, and (d) within 60 min after smoking. Exhaled breath was collected by a previously validated method and analyzed by an e-nose (Cyranose 320). By principal component analysis, significant variations in the exhaled VOC profile were shown for principal component 1 and 2 before and after smoking. Significance was higher 30 and 60 min after smoking than 5 min after (*p* < 0.01 and <0.05, respectively). Canonical discriminant analysis confirmed the above findings (cross-validated values: baseline vs. 5 min = 64.6%, AUC = 0.833; baseline vs. 30 min = 83.6%, AUC = 0.927; baseline vs. 60 min = 89.6%, AUC = 0.933). Thus, the exhaled VOC profile is influenced by very recent smoking. Interestingly, the effect seems to be more closely linked to post-cigarette inflammation than the tobacco-related odorants.

## 1. Introduction

Breathomics is the study of exhaled breath composition, which could ease the diagnosis and phenotyping of several respiratory diseases [1]. Exhaled breath contains thousands of volatile organic compounds (VOCs), including exogenous VOCs such as environmental compounds, food, drinks, and drugs and endogenous VOCs such as metabolic derivates and microbiota. Basically, any changes occurring in the cell biochemistry, including those caused by pathologies, can change the blood composition and can be reflected in breath by the interchange of VOCs in the lungs [2]. Breath is a less complex medium compared to blood, stool, and urine, making its sampling and/or data analysis less complicated [2].

Gas chromatography–mass spectrometry (GC–MS) is the gold standard for measuring exhaled breath VOCs. However, GC–MS is a cumbersome and expensive instrument and requires well-trained personnel; thus, its application in breath analysis is limited [1]. 

The electronic nose (e-nose) is a quick and non-invasive tool which can detect exhaled VOC profiles. Interestingly, an increasing number of studies have shown its potential in various respiratory diseases, including asthma, COPD, lung cancer, pneumonia, interstitial lung disease [1], and even non-respiratory conditions such as breast and colon cancer, neurodegenerative diseases, diabetes mellitus, and liver failure [3]. 

Despite being a promising diagnostic technique, before obtaining full validation of exhaled breath profiling, there are still several methodological limitations due to different factors such as smoking, diet, race, physical exercise, pregnancy, and medication use, which may have an impact on the exhaled VOC composition [4].

Among the aforementioned factors, smoking plays a crucial role, since VOCs derive from metabolic and inflammatory processes related to physiological and/or pathophysiological changes occurring in the respiratory tract [5], and smoking may contribute to the alteration of these processes [6]. Moreover, more than 90 VOCs are known to be present in the mainstream smoke of cigarettes, including nitrogenous compounds, aldehydes, esters, alkenes, aromatic hydrocarbons, ketones, alcohols, furfurans, acids, alkanes, and ethers, which have well-known injurious and carcinogenic effects [7,8].

To date, it remains unclear how exhaled VOCs are influenced by smoking status. Based on the above, the aim of our study was to assess whether an e-nose can discriminate exhaled breath before and after smoking at different time periods in a group of healthy volunteers. 

## 2. Materials and Methods

### 2.1. Patients

A total number of 24 healthy smokers (11 males, 13 females) were enrolled in our study. All participants had a negative clinical history of chest symptoms and/or systemic diseases, and none of the subjects were taking any medications. The age range was 24–47. All individuals had a normal lung function. Subjects with upper or lower respiratory tract infections in the four weeks before testing were excluded from the study. Baseline characteristics are shown in Table 1.

Exhaled breath was collected from all individuals as follows: (a) before smoking (T0), (b) within 5 min after smoking (T1), (c) within 30 min after smoking (T2) and (d) within 60 min after smoking (T3).

All participants were volunteers and were enrolled from hospital staff relatives.

The current study was previously approved by the local ethics committee (protocol number 46403/15), and all individuals were required to sign an informed consent form before participating in the current study.

### 2.2. Study Design

A longitudinal study was performed. Two separate visits were required to complete all measurements. During visit one, all participants were judiciously screened for inclusion/exclusion criteria and, after definitive inclusion in the study, flow-volume spirometry was executed (MasterscreenPneumo, Jaeger, Würzburg, Germany).

During visit two, exhaled breath was collected before smoking (T0) and after smoking one cigarette (tar content: 10 mg, nicotine level: 0.8 mg, carbon monoxide level: 10 mg) at T1, T2, and T3 as described above and immediately analyzed by the e-nose. All participants were asked not to smoke at least 12 h prior to the test. Subjects were asked to refrain from eating and drinking, as well as from performing active physical activity, for at least 3 h before visit two. Exhaled breath was collected as previously validated [6,7]: first, a 5-min wash-in period by a 3-way non-rebreathing valve connected to an inspiratory VOC filter (A2; North Safety, Middelburg, the Netherlands) to minimize the impact of environmental VOCs and to an expiratory silica reservoir to reduce the effect of humidity on sensors (which are very sensitive to H_2_O). Subsequently, participants exhaled into a Tedlar bag connected to the e-nose until approximately reaching their residual volume. A nose clip was worn during all the maneuvers. All participants were remotely monitored by text messages or phone calls for any kind of symptoms within 24 h from breath collection. None of the participants experienced hyperventilation or other related symptoms during breath collection and in the following 24 h.

### 2.3. Electronic Nose

**Figure 1:**
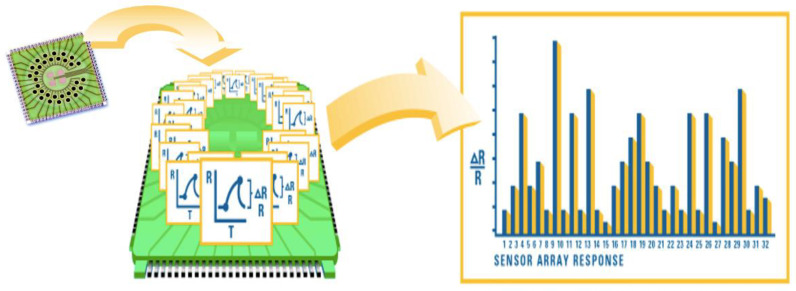
Working principle of Cyranose 320: it is based on a nano-composite array of 32 organic polymer sensors. If exposed to VOC combinations, the polymers swell, thereby modifying their electrical resistance. Raw data are registered as the increase in resistance of any single sensors and the combination of all signals results in a “breathprint”.

A commercially available e-nose was used for the current study (Cyranose 320, Sensigent, Irwindale, CA, USA). It is based on a nano-composite array of 32 organic polymer sensors. If exposed to VOC combinations, the polymers swell, thereby modifying their electrical resistance. Raw data are registered the as increase in resistance of any single sensors in an onboard built database, resulting in a “breathprint” which labels the VOC spectrum and that can be successively used for pattern-recognition algorithms (Figure 1). According to the instruction manual, we used the following operating parameters: baseline purge: 30 s (pump speed: low); sampling time: 60 s (pump speed: medium), purging time: 200 s (pump speed: high), total run time: 300 s, temperature 42 °C. Post-run purges between samples: 5 min. Moreover, before the first sample of the day, 5 min exposure to room air followed by a “blank measurement” was performed to stabilize sensor outputs. Relative humidity for exhaled samples was around 55%.

### 2.4. Statistical Analysis

All data analyses were performed using SPSS for Windows 26.0 (SPSS, Chicago, IL, USA). The normal distribution of our data was verified by Kolmogorov–Smirnov and Shapiro–Wilk tests. We analyzed categorical values with the Fisher’s exact test or χ^2^ test as appropriate and reported as n (%). We compared continuous variables by ANOVA and Student’s *t*-test for independent samples. Continuous parameters with normal distribution were reported as mean ± standard deviation (SD). 

We performed data reduction of the whole set of 32 sensors by principal component analysis. All 4 principal components (PCs) were compared at different times (before smoking, 5 min after smoking, 30 min after smoking, and 60 min after smoking) by ANOVA test. When significant, the individual groups were compared 2 to 2 with Student’s *t*-test for independent samples. 

The significant principal components were analyzed by linear discriminant analysis (CDA) for categorizing VOC patterns. The “leave-one-out” validation method was used to calculate the cross-validated accuracy % (CVA%), which estimates the accuracy of a predictive model in practice. Additionally, a receiver operating characteristic curve (ROC curve) was built using predicted probabilities to determine the area under the curve (AUC). The sample size was estimated to limit the standard error to 10%. Assuming 80% accuracy, the current sample size per subgroup sufficed. We considered a *p*-value of <0.05 as statistically significant.

## 3. Results

The characteristics of the study population are described in Table 1. Males and females were almost equal, and the lung function as well as body mass index (BMI) were within ranges of normality. 

**Figure 2:**
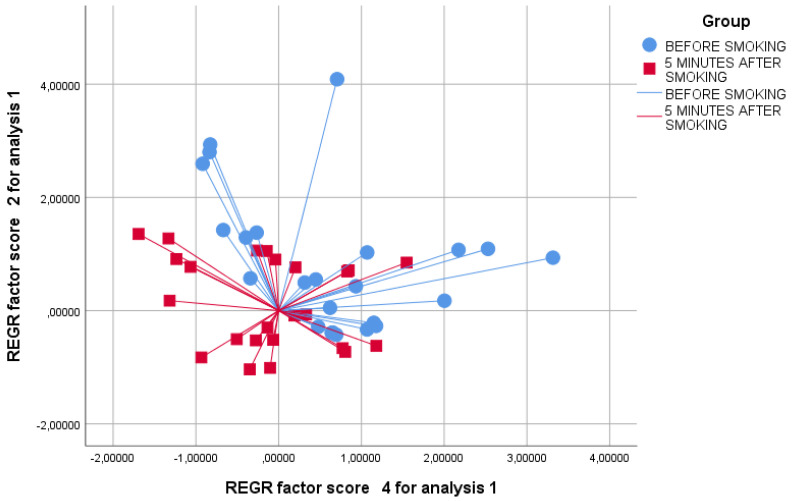
Two-dimensional PCA plot showing the discrimination of breathprints before smoking (blue circles) and 5 min after smoking (red squares). REGR = regression.

**Figure 3:**
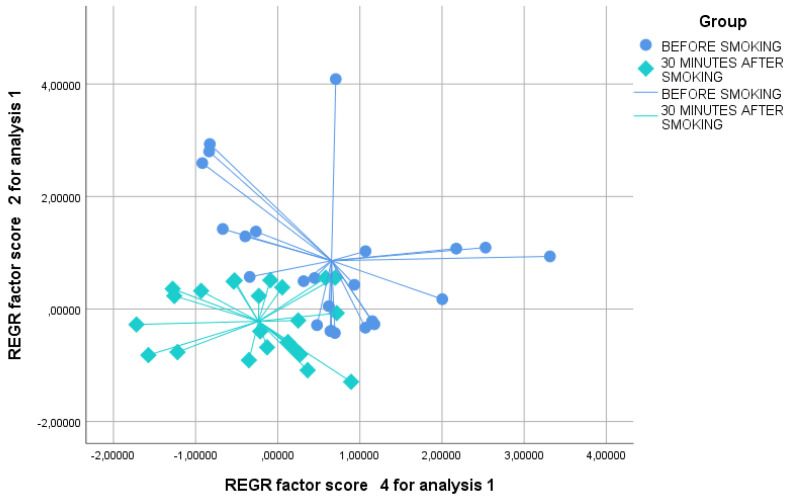
Two dimensional PCA plot showing the discrimination of breathprints before smoking (blue circles) and 30 min after smoking (green diamonds). REGR = regression.

**Figure 4:**
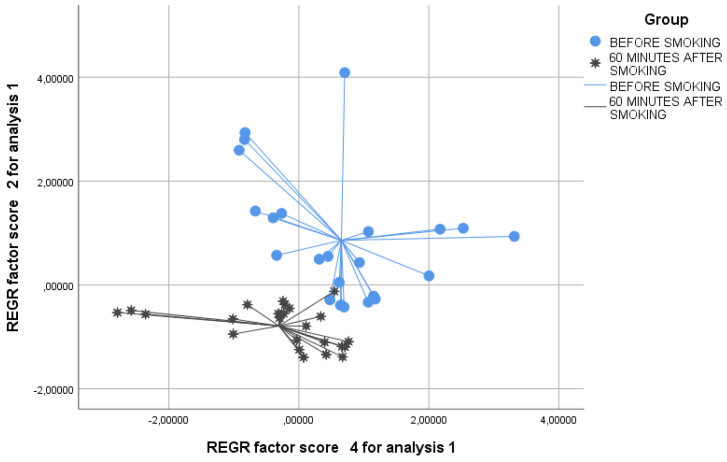
Two-dimensional PCA plot showing the discrimination of breathprints before smoking (blue circles) and 60 min after smoking (black stars). REGR = regression.

**Figure 5:**
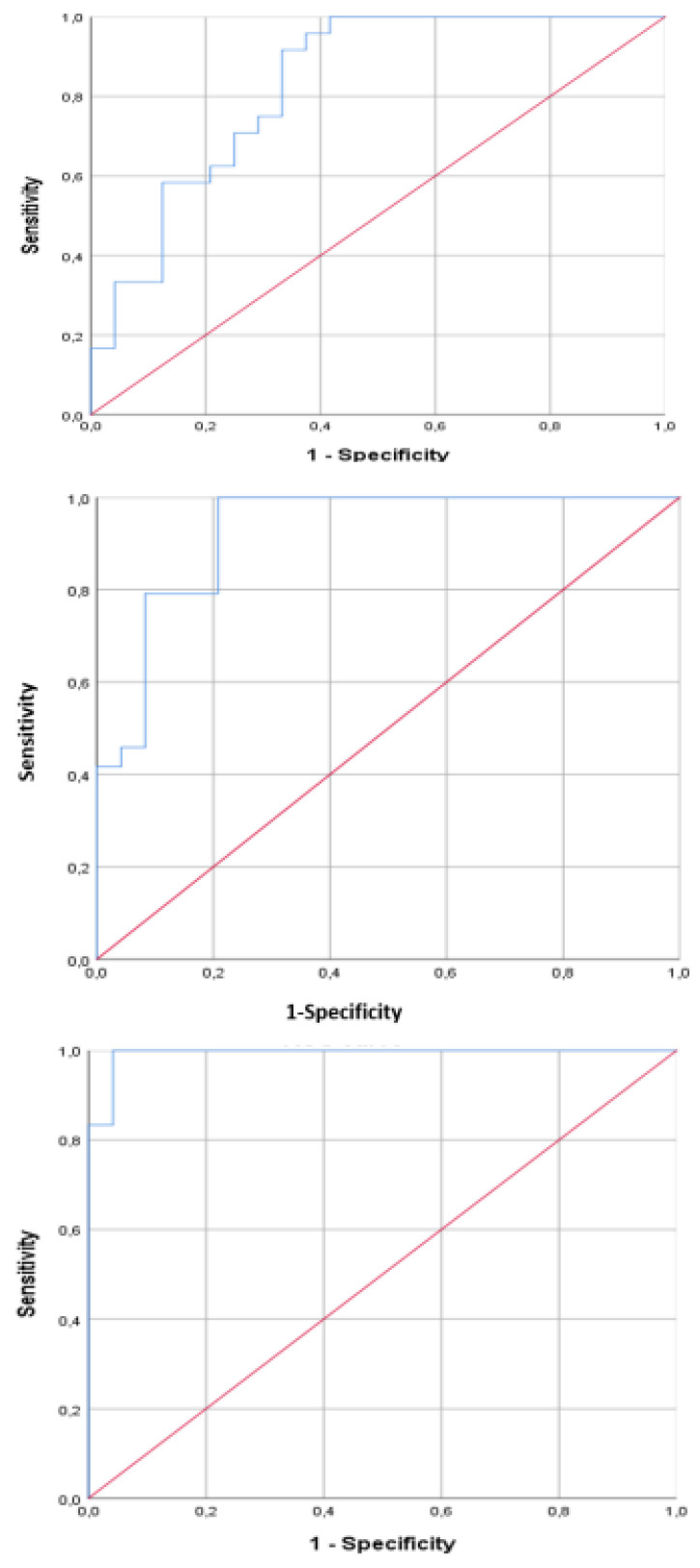
ROC curves between T0 and T1 (**upper**), between T0 and T2 (**center**), and between T0 and T3 (**lower**).

We verified by the Kolmogorov test that the principal component had a normal distribution. By principal component analysis (PCA), significant variations in the exhaled VOC profile were shown for PCs 2 and 4 before and after smoking (Table 2); hence, we selected these two factors for further analysis. Interestingly, significance was higher 30 and 60 min after smoking than 5 min after (T0 vs. T2 and T0 vs. T3, *p* < 0.01 and T0 vs. T1 *p* < 0.05, see Table 3). The two-dimensional PCA plot showed the discrimination of breathprints before smoking and 5 min after smoking (Figure 2). Similarly, the 2-D PCA plot showed the distinction of breathprints before smoking and 30 min after smoking (Figure 3). Finally, the discernment of breathprints before smoking and 60 min after smoking was also shown (Figure 4). Subsequent linear discriminant analysis confirmed the above findings with the following cross-validated values: T0 vs. T1 = 64.6%, T0 vs. T2 = 83.6% and T0 vs. T3 = 89.6%. The area under the curve of the ROC curve for the discrimination between exhaled VOC profiles before and after smoking were the following: T0 vs. T1 = 0.832, T0 vs. T2 = 0.927 and T0 vs. T3 = 0.933 (Figure 5).

## 4. Discussion

In our study, we demonstrated that the use of an e-nose to analyze the exhaled VOC profiles is influenced by very recent smoking. Interestingly, the effect seems to be more closely linked to the post-cigarette inflammation than the tobacco-related odorants.

E-nose-based exhaled breath analysis has been increasingly used in recent years for various pathologies for screening and diagnostic purposes [1,9]. Breath analysis might be preferrable compared to other biological samples such as blood, feces, and urine since breath is a totally non-invasive matrix, much easier to acquire, and has the potential to offer real-time monitoring [2].

The novelty of our study is the assessment of e-nose analyzed exhaled breath VOC composition in relation to short-term smoke exposure in a population of well-characterized, healthy subjects.

The strengths of our study are the careful selection of our population of smokers, with the exclusion of any known diseases and the use of standardized methods for e-nose analysis, with all participants smoking the same type of cigarette. On the other hand, a few limitations must be disclosed. The first is our relatively small number of enrolled subjects. However, based on previous observations, [10,11] and based on our sample size estimation, we believe that our total population of 24 individuals might warrant further investigations including larger cohorts and a validation group.

Second, we arbitrarily chose sampling periods after smoking for each group and we lack data on longer periods after smoking; therefore, we may have missed some relevant information.

Third, although being non-invasive, easy to use, and with promptly available results, e-nose analysis does not allow the identification and quantification of single VOCs. Incontestably, further investigations should integrate chemical analytical techniques such as gas chromatography–mass spectrometry (GC–MS) to identify discriminant VOCs.

How can we interpret our data?

Tobacco smoke is known to directly affect the level of several VOCs in human breath [12]. Nonetheless, previous studies on exhaled breath analyzed by an e-nose indicate that signals most likely derive from pathophysiological modifications driven by the underlying chronic airway disease and by chronic smoking exposure. In detail, a recent investigation by Principe et al. [13] showed that an e-nose could discriminate ever- from never-smokers in a population of subjects with COPD and asthma. However, their e-nose could not distinguish recent smokers as effectively, denoting that recent smoking might not be a confounding factor of the VOC spectrum [13].

Similarly to the above, previous studies with e-nose recruiting smoking or ex-smoking patients with asthma and COPD concluded that several types of e-nose can discriminate among patients with chronic smoking habits, whereas it cannot detect active smoking in patients according to the time of last cigarette consumption [14,15,16,17,18,19,20].

Our data are in line with the above observations; thus, we may speculate that e-nose detects the effect of cigarette smoking on airways rather than the smoke itself.

## 5. Conclusions

In conclusion, our data indicate that very short-term smoking may be considered as a confounder which affects e-nose measurements. Therefore, we suggest that patients should abstain from smoking for a certain period before testing their exhaled breath for e-nose-based VOC analysis. Future studies with larger cohorts and with different types of e-nose technology should be addressed to extend our findings and to explore other confounding factors.

## Figures and Tables

**Table 1:** Clinical characteristics of the studied population.

Parameter	Value
Subjects (n.)	24
M/F (n.)	11\13
Age (y.)	35.4 ± 11.3
FEV1%pred.	101.5 ± 11.8
BMI (kg/m^2^)	25.77 ± 3.2
Current smokers (n.)	24
Comorbidities (n.)	0

FEV1 = forced expiratory volume in the 1st second.

**Table 2:** ANOVA of principal components at different time periods.

	PC1	PC2	PC3	PC4	p
**T0**	−0.259 ± 0.499	0.858 ± 1.215	−0.210 ± 0.723	0.654 ± 1.092	0.114
**T1**	−0.009 ± 0.644	0.151 ± 0.800	0.023 ± 1.004	−0.114 ± 0.854	0.000
**T2**	0.402 ± 0.506	−0.218 ± 0.609	0.381 ± 0.947	−0.235 ± 0.753	0.140
**T3**	−0.133 ± 1.718	−0.790 ± 0.382	−0.193 ± 1.209	−0.305 ± 1.012	0.002

**Table 3:** Discriminant analysis by means of PC2 and PC4 between before smoking and after smoking time periods.

Time	Cross Validate Value (%)	AUC [CI]; *p* Value
T0 vs. T1	64.6	0.832 [0.715–0.948]; *p* < 0.05
T0 vs. T2	83.6	0.927 [0.853–1.000]; *p* < 0.01
T0 vs. T3	89.6	0.933 [0.977–1.000]; *p* < 0.01

## Data Availability

Not applicable.
